# Impact of modelling choices on setting the reference levels for the EU forest carbon sinks: how do different assumptions affect the country-specific forest reference levels?

**DOI:** 10.1186/s13021-019-0125-9

**Published:** 2019-09-03

**Authors:** Nicklas Forsell, Anu Korosuo, Mykola Gusti, Sebastian Rüter, Petr Havlik, Michael Obersteiner

**Affiliations:** 1 International Institute for Applied Systems Analysis (IIASA), Schlossplatz 1, 2361 Laxenburg, Austria; 2 Lviv Polytechnic National University, 12 Bandera Str., Lviv, 79013 Ukraine; 3 Thünen Institute of Wood Research, Leuschnerstraße 91c, 21031 Hamburg, Germany

**Keywords:** Forests, Reference levels, LULUCF, EU LUUCF regulation, Carbon accounting, Forest management, G4M, WoodCarbonMonitor

## Abstract

**Background:**

In 2018, the European Union (EU) adopted Regulation 2018/841, which sets the accounting rules for the land use, land use change and forestry (LULUCF) sector for the period 2021–2030. This regulation is part of the EU’s commitments to comply with the Paris Agreement. According to the new regulation, emissions and removals for managed forest land are to be accounted against a projected forest reference level (FRL) that is estimated by each EU Member State based on the continuation of forest management practices of the reference period 2000–2009. The aim of this study is to assess how different modelling assumptions possible under the regulation may influence the FRL estimates. Applying the interlinked G4M and WoodCarbonMonitor modelling frameworks, we estimate potential FRLs for each individual EU Member State following a set of conceptual scenarios, each reflecting different modelling assumptions that are consistent with the regulation and the technical guidance document published by the European Commission.

**Results:**

The simulations of the conceptual scenarios show that differences in the underlying modelling assumptions may have a large impact on the projected FRL. Depending on the assumptions taken, the projected annual carbon sink on managed forest land in the EU varies from −319 MtCO_2_ to −397 MtCO_2_ during the first compliance period (2021–2025) and from −296 MtCO_2_ to −376 MtCO_2_ during the second compliance period (i.e. 2026–2030). These estimates can be compared with the 2017 national GHG inventories which estimated that the forest carbon sink for managed forest land was −373 MtCO_2_ in 2015. On an aggregated EU level, the assumptions related to climate change and the allocation of forest management practices have the largest impacts on the FRL estimates. On the other hand, assumptions concerning the starting year of the projection, stratification of managed forest land, and timing of individual management activities are found to have relatively small impacts on the FRL estimates.

**Conclusions:**

We provide a first assessment of the level of uncertainty associated with the different assumptions discussed in the technical guidance document and the LULUCF regulation, and the impact of these assumptions on the country-specific FRL. The results highlight the importance of transparent documentation by the EU Member States on how their FRL has been calculated, and on the underlying assumptions.

## Background

### EU LULUCF Regulation

In June 2018, the European Union (EU) adopted Regulation 2018/841, which sets the accounting rules for the land use, land use change and forestry (LULUCF) sector in the EU for the period 2021–2030 [1]. This LULUCF Regulation is part of the EU’s commitments to comply with the Paris Agreement, where the parties agreed to limit global temperature increase to well below 2 °C above pre-industrial levels, and to pursue efforts to limit this increase to 1.5 °C [2]. The Paris Agreement urges its Parties to preserve and enhance existing carbon sinks including forests, bringing the LULUCF sector and forests for the first time an integral part of international climate mitigation targets. Through its LULUCF Regulation, the EU became the first party to announce its accounting system for the LULUCF beyond the Kyoto Protocol, which will be discontinued after 2020.

In its 2030 Climate and Energy Framework, the EU has committed to decrease its net greenhouse gas (GHG) emissions by 40% by 2030, compared to the emission level in 1990 (European Commission, 2016). This target is set economy-wide, and it is further divided into different targets for different sectors. The LULUCF sector, which has historically been a net carbon sink in the EU, may contribute to the target through a transfer of “credits”, i.e. additional carbon savings, to counterbalance needed savings on the so-called Effort Sharing sector (road transport, buildings, waste, agriculture). As stipulated in the Effort Sharing Regulation 2018/842 [3], these credits may be a maximum of 280 MtCO_2_e per year on the level of the whole EU. In addition, the LULUCF regulation sets a no-debit rule for the LULUCF sector, meaning that in the EU as a whole, the accounted emissions on the LULUCF sector may not exceed the sector’s accounted removals.

Although this is the first time that the LULUCF sector is accounted for within the overarching EU 2030 Climate and Energy Framework, the emissions and removals of the sector have been tracked in the national GHG inventories since 1990. The GHG inventories submitted to the United Nations Framework Convention on Climate Change (UNFCCC) serve also as a basis for the categories considered in the LULUCF Regulation. The LULUCF Regulation accounts for emissions and removals from afforestation and deforestation using gross-net accounting, i.e. accounting for the total emissions and removals on afforested and deforested land [1, Art 6]. The emissions and removals from other land categories are accounted for using a net–net approach: comparing the emissions and removals of anthropogenic activities to a base year or a base level [1, Art 7 and Art 8]. For managed cropland, managed grassland and managed wetlands, the emissions and removals are compared to a historical base period of 2005–2009 [1, Art 7]. For managed forest land, on the other hand, accounting is based on a comparison against a projected reference level [1, Art 8]. This forest reference level (FRL) projects the continuation of forest management practices of the reference period (RP) 2000–2009, over the two compliance periods covered by the LULUCF Regulation, 2021–2025 and 2026–2030 [1, Art 8.1]. This projected FRL will then be used as a baseline against which the realized emissions and removals on managed forest land will be compared with.

A reference level has been used to account for emissions and removals from forest management already under the second commitment period of the Kyoto Protocol (2013–2020), and is also used as a tool to account for forest emissions and removals under the REDD+ regime [4, 5]. The reference level provides a means of considering the long time-horizon and legacy effects of past management practices that are usually associated with forestry. The purpose is to ensure that the accounting only takes account of the changes in the carbon balance that occur because of human actions since the reference period, and not because of emissions or removals that occur purely because of the natural aging of the forests, or because of effects of historical management or natural disturbances in the past [6].

### Calculation of the FRL

The FRL is a projection of GHG emissions and removals on managed forest land, showing what the emissions and removals would be if the past management was continued without changes. The carbon pools included in the FRL are above- and below-ground biomass, litter, dead wood, soil organic carbon and harvested wood products (HWP) [1, Section B. of Annex I]. According to the LULUCF Regulation, the FRL “*shall be based on the continuation of sustainable management practice, as documented in the period from 2000 to 2009 with regard to dynamic age*-*related forest characteristics in national forests, using the best available data*” [1, Art 8.5]. In addition, there is a number of other criteria that need to be complied with, such as the need to ensure consistency with the GHG inventories and a robust and credible accounting system, consistency with the objective of sustainable use of natural resources, as well as being consistent with the goal of the Paris Agreement to achieve a balance between the anthropogenic emissions by sources and removals by sinks by the second half of this century [1, Section A. of Annex IV].

The FRL is estimated by each individual EU Member State and reported as a part of a National Forestry Accounting Plan (NFAP), which also describes the Member State’s long-term forest strategy and possible scenarios foreseen for the forest sector. Each Member State was required to submit their proposed FRL for the period 2021–2025 to the European Commission by the end of 2018 [1, Art 8.3]. The submissions are reviewed by the Commission and Member State-nominated experts during 2019, with the final adoption of the delegated acts set to the end of October 2020 [1, Art 8.6 and 8.8].

Although the LULUCF Regulation builds on the previous experiences of accounting under the Kyoto Protocol, there are also many differences and updates compared to the previous requirements. The forest management reference level (FMRL) under the Kyoto Protocol was developed using assumptions on the future effects of policies in the business-as-usual scenario. Consequently, this approach allowed an inclusion of assumptions of increasing forest harvest—and therefore—decreasing forest sinks through expectations on increasing forest use in the future [7]. Grassi et al. [7] suggest that there is indication of overestimating the emissions in the FMRL through unrealistic assumptions on future impact of policies. When comparing the realized GHG net emissions against the FMRL, some actions that were foreseen to reduce the forest sink may not have been realized. Based on these experiences, the accounting rules for the FRL were further developed in the LULUCF Regulation. Most importantly, the FRL under the LULUCF Regulation does not include assumptions on future impact of policies. Instead, it is meant to be strictly based on the continuation of past management practices, while allowing for the development of the forest structure through age-related dynamics only [8].

The LULUCF regulation sets out several criteria and lead statements for the estimation of the FRLs in the Member States, including requirements of continuation of past forest management practices, completeness of the accounts, and consistency with the methods and results of the GHG inventories. However, forest modelling requires a myriad of assumptions for which the LULUCF regulation does not provide specific requirements. Moreover, the Member States have very different conditions in terms of forest characteristics, model setups, as well as previous experiences with forest modelling for carbon accounting. For the second commitment period of the Kyoto Protocol, the European Commission provided strong technical support for the preparation of the FMRLs, and the countries could choose to either prepare their own estimates or entrust a modelling team using a large-scale model capable of projecting the estimates for all EU Member states. In the end, ten Member States submitted FMRL estimates based on country-specific methodologies, while 15 Member States used estimates based on the large scale G4M [9], EFISCEN [10, 11]; and the WoodCarbonMonitor [12] models, and two Member States reported a linear extrapolation of historical emissions data (1990–2008) due to insufficient data available for modelling [13]. The approach used in the joint G4M-EFISCEN-WoodCarbonMonitor projections is detailed in Böttcher et al. [14] and Rüter [15]. For the FRL under the LULUCF Regulation, however, each Member State is responsible for calculating and reporting their national FRL.

Modelling always requires assumptions, and due to the diversity in national circumstances, certain assumptions may have more impact on the results than others. While the LULUCF Regulation is strict in certain aspects, it leaves open many technical details that need to be decided for the projection of the FRL. A technical guidance document [8] was published by the European Commission to assist the countries in preparing their FRLs. The technical guidance document discusses several different assumptions and methods to consider when preparing the FRLs that are necessary for the FRL modelling but not explicitly covered by the LULUCF Regulation. Such considerations are for instance whether the projection for 2021–2030 should use the latest inventory data or rather continue right from the reference period (in this case, the data for 2010 would already be a modelling result, and possibly differ from the realized situation for 2010); how to consider possible trends observed during the reference period; and whether the projections should include impacts of climate change on forest growth and yield.

A first methodological approach of the FRL and assessment of the potential results for 26 EU Member States (EU28 excluding Cyprus and Malta) was presented by Grassi et al. [7]. Their approach is fully in line with the advice provided in the technical guidance document published by the European Commission and the methodology was included as one of the possible alternatives that the individual Member States could use to estimate their country-specific FRLs. However, the sensitivity of the FRLs to changes in key modelling assumptions or data sources was not assessed. A second assessment of potential FRLs for 26 EU Member States (EU28 excluding Cyprus and Malta) and their related impacts on total wood harvest levels was presented by Nabuurs et al. [16]. In that assessment, the EFISCEN model [10, 11] was utilized to calculate the impacts of three scenarios that reflect different interpretations of the LULUCF regulation as put forward by the authors. However, all of these scenarios are not in line with the advice provided in the technical guidance document published by the European Commission, nor does the approach consider all major carbon pools as only the living biomass carbon pools are considered. As such, it is not known to what extent the country-specific FRL may be influenced by the different assumptions allowed for by the LULUCF regulation and the associated technical guidance document published by the European Commission. For the credibility of the approach and for the Member States’ FRLs to be assessed in a consistent manner, it is vital to know which modelling assumptions may have a significant influence on the country-specific FRL, and which assumptions are likely to have relatively small impact on the FRL.

### Aim of this study

The aim of this study is to assess the consequences of different modelling assumptions, that are possible under the regulation, on the EU Member States’ national FRLs under the LULUCF regulation. We estimate the country-specific FRLs in a consistent manner for all EU Member States based on a set of conceptual scenarios. Each scenario reflects a different interpretation of the LULUCF Regulation, but they are all still fully in line with the advice provided in the technical guidance document published by the European Commission. As such, all scenarios are based on the key requirement in the LULUCF regulation that the FRL shall be estimated based on the continuation of forest management practice of the reference period, with regard to the age-related dynamics in the forests. Applying the interlinked G4M [9] and WoodCarbonMonitor [12] modelling frameworks, we estimate country-specific FRLs that cover emissions and removals from the major carbon pools as specified in the LULUCF Regulation, including above and below-ground biomass and harvested wood products (HWP). Other carbon pools covered by the LULUCF Regulation (litter, dead wood and soil organic carbon) are assumed to remain constant as their development currently cannot be projected by the modelling frameworks. Both models apply an annual time step, making them highly suitable for this type of an analysis. Through scenario analysis, we assess the uncertainty associated with the FRL estimates and highlight how different modelling assumptions may influence the projected emissions and removals (net carbon sink) for managed forest land in the EU.

## Results

### Scenarios for estimating the forest reference level

To analyze how the flexibility associated with the LULUCF Regulation may impact the country-specific FRLs and the projected net carbon sink in managed forest land, a total of 12 conceptual scenarios were developed and estimated utilizing the interlinked G4M and WoodCarbonMonitor modelling frameworks. Each of these scenarios are estimated in accordance with the LULUCF Regulation [1] and the technical guidance document published by the European Commission [8]. Most of these scenarios have been constructed directly based on the different alternatives for estimating the FRL provided in the technical guidance document.

The 12 conceptual scenarios were developed by combining different assumptions across the following key parts of the modelling:

- Starting year for the projection of the FRL. Here, assumptions are tested concerning the year in which the projections of the FRL is started (i.e. 2010 or 2015) and from which the state of the forest is estimated as an output of the modelling framework.
- The level of detail to which the area of managed forest land is stratified or divided into categories with different management. Here, different levels of stratification are tested for two key criteria: stratification according to (i) tree species, and (ii) forest growing conditions.
- Assumptions concerning the spatial allocation of forest management practices (FMPs). Here, the FRL is calculated based on different assumptions used to allocate two general categories of forest management practices to the area of managed forest land. These two categories of FMPs are: (i) forest management practices with clearcutting, (ii) and forest management practices without clearcutting.
- Assumptions related to the timing of individual management activities. A forest management practice can be defined as a set of silvicultural operations being carried out at different phases of the stand development. Here, we assess three different criteria defining when clearcutting should take place: (i) the average timing as documented during the reporting period, (ii) the latest documented value (i.e. 2009 or 2014 depending on starting year), (iii) or a combination of the two.
- Assumptions concerning climate change. Here, the FRL is calculated based on two different assumptions: no consideration to impact of climate change on forest growth, and based on a projection of future climatic conditions and accounting for the related changes in the growing conditions.

An overview of the scenarios and their key assumptions can be found in Table 1 and a detailed description of the assumptions for each scenario is provided in “Methods” section. All of these specific assumptions are examples of technical choices that need to be made in the beginning of the modelling exercise, but for which the LULUCF regulation does not provide further specification. As it is not possible to judge which alternative assumption is preferable over another under the LULUCF regulation, we chose to not choose a single scenario as a reference point, but instead modelled different combinations of assumptions, and analyze the results in the view of these assumptions. It should be noted that a number of additional alternatives for estimating the FRL are also mentioned in the technical guidance document but are not assessed within this study (for example, modelling of natural disturbances and the assumptions on the future development of managed forest land area).

**Table 1:** Overview of the scenarios as applied to assess the uncertainty associated with different modelling assumptions for estimating the FRL

Scenario name	A	B	C	D	E	F	G	H	I	J	K	L
Starting year	2010	2015	2010	2010	2010	2010	2010	2010	2015	2015	2015	2015
Stratification	Full	Full	1MAI	1Species	1MAI and 1Species	Full	Full	Full	Full	Full	Full	Full
Allocation of FMPs	Average	Average	Last	Last	Last	Last	Last	Last	Last	Last	Last	Last
Timing of activities	Average	Average	Average	Average	Average	Average	Latest	Combined	Average	Latest	Combined	Average
Climate change	No	No	No	No	No	No	No	No	No	No	No	Yes

The scenarios are not arranged in any particular order, but instead provide an array of different alternative assumptions possible in the modelling of the FRL. A detailed description of the scenario-specific modelling assumptions is provided in “Methods” section

### Impact of the scenarios on the EU sink

**Fig. 1:**
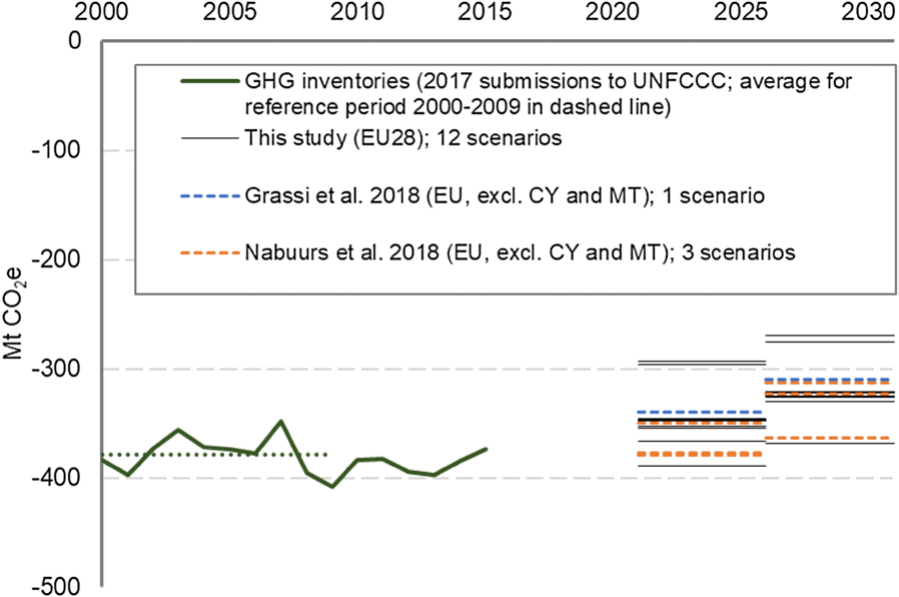
The net forest carbon sink in the FRL (excluding HWP) at the aggregated EU28 level. The results of this study are shown as the average sink for the first and second compliance periods. The estimates of this study are compared with GHG inventories submitted by the Member States to the UNFCCC (2017), and scientific assessments by Grassi et al. [7] and Nabuurs et al. [16]. In Grassi et al. [7] and Nabuurs et al. [16], only 26 EU Member States were considered (EU28 excluding Cyprus and Malta). Grassi et al. [7] consider all carbon pools (here shown excluding HWP), while in Nabuurs et al. [16] only the living biomass pools are accounted for. It should also be noted that there are differences in the underlying scenario assumptions and data sources between this study and the analyses of Grassi et al. [7] and Nabuurs et al. [16]

Utilizing the interlinked G4M and WoodCarbonMonitor modelling frameworks, we estimated country-specific FRLs for each EU Member State for the 12 conceptual scenarios. Figure 1 shows the net forest carbon sink^1^ (excluding HWP) at the aggregate EU28 level for the different FRLs and how they compare to national GHG inventories [17] and other publicly available estimates [7, 16]. In 11 out of the 12 scenarios, the net forest carbon sink is projected to decline (i.e. the sink decreases) as compared to current level for the first compliance period (2021–2025) and further decline for the second compliance period (2026–2030). According to the national GHG inventories submitted by the Member States to the UNFCCC (2017), the forest carbon sink was −373 MtCO_2_ for EU28 in 2015. In this assessment, it is projected that under the continuation of the management practice of 2000–2009, the forest carbon sink would be in the order of −319 to −397 MtCO_2_ during the first compliance period, and −296 to −376 MtCO_2_ during the second compliance period (see Fig. 1). It should be noted that a decline of the forest carbon sink is consistent with previous projections in the scientific literature [7, 16, 18]. The expected decline of the net forest carbon sink between the compliance periods is related to the anticipated increase of the forest harvest level (see Table 2) due to aging forests and also reflects the recent trend of slightly declining EU total stem volume increments^2^ that has been reported in forest inventories [18]. This reduction in the increment also explains why our and other published estimates project that the net forest carbon sink will decrease further from the first to the second compliance periods. In our estimates, the aggregate EU28 net forest carbon sink is projected to be on average 22 MtCO_2_ (21 to 36 MtCO_2_) smaller in the second compliance period than in the first compliance period, depending on the scenario (see Table 2).

**Table 2:** Annual harvest level and net forest carbon sink (excluding HWP) for EU28 in the different scenarios for the reference period 2000–2009, and in the projected FRL during the two compliance periods

Scenario	Roundwood harvest per year [million m^3^ over bark]			Net forest carbon sink (excluding HWP) [MtCO_2_e]		
	Reference period, average (2000–2009)	Compliance period 1 (2021–2025)	Compliance period 2 (2026–2030)	Reference period, average (2000–2009)	Compliance period 1 (2021–2025)	Compliance period 2 (2026–2030)
A	474	505	512	−379	−293	−270
B	474	505	510	−379	−296	−276
C	474	463	475	−379	−353	−323
D	474	465	477	−379	−355	−326
E	474	457	475	−379	−366	−330
F	474	465	475	−379	−348	−322
G	474	466	476	−379	−347	−322
H	474	466	476	−379	−347	−322
I	474	468	475	−379	−348	−326
J	474	469	475	−379	−346	−325
K	474	469	475	−379	−346	−326
L	474	442	457	−379	−389	−368

**Fig. 2:**
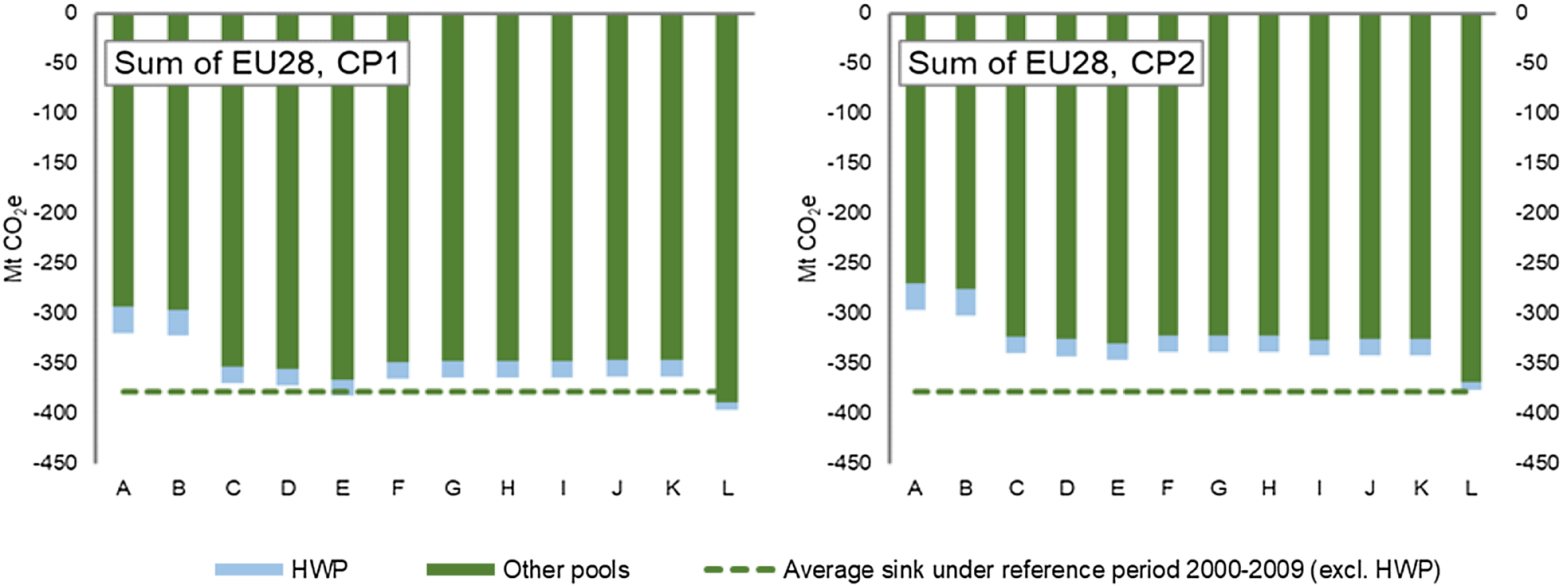
The aggregate EU28 FRL (sum of country-specific FRLs) during the first compliance period (CP1) and second compliance period (CP2) in the different scenarios. ‘Other pools’ include above and below-ground biomass (deadwood, litter and soil organic carbon are assumed to remain constant)

The different prospective scenarios for calculating the FRLs show very different outcomes in terms of the projected net forest carbon sink. Depending on the scenario, the EU28 aggregated forest carbon sink (including HWP) varies with as much as 78 MtCO_2_ (−319 to −397 MtCO_2_) for the first compliance period, and with 80 MtCO_2_ (−296 and −376 MtCO_2_) for the second compliance period. Figure 2 shows the aggregate EU28 forest carbon sink for the first and second compliance periods, including and excluding HWP. At the EU28 level, relatively small variations in the estimated net forest carbon sink (0.2 to 9.7 MtCO_2_ and 0.1 to 4.8 MtCO_2_ for the first and second compliance periods, respectively) can be seen between a majority of the scenarios (i.e. scenarios C, D, F, G, H, I, J and K). However, three scenarios in particular stand out, these being scenarios A, B, and L.

Scenarios A and B project clearly the smallest net forest carbon sink for both first and second compliance periods. The main underlying reason for this finding is that these scenarios estimate the highest forest harvest levels (see Table 2) during both compliance periods. In scenarios A and B, the area of land allocated to timber production with/without clearcutting is estimated based on the average over the reference period. In the other scenarios, allocation of each forest management practice is based on the last year of the reference period, i.e. the same as in 2009. During the reference period, the annual EU28 forest harvest level first increased from 2001 to 2007, and then declined sharply from 2008 to 2009 due to a downturn in the EU economy [19]. A higher harvest level is associated in G4M with more forest being used for timber production with clearcutting, while a lower harvest level has proportionally more forest area used for timber production without clearcutting. Assuming the *average* approach to allocate forest management practices in scenarios A and B (see Table 1) results therefore in more forest area allocated to timber production with clearcutting, than in the other scenarios which assume the continuation of the *last* allocation of forest management practices (i.e. that the area allocation as of 2009).

On the contrary, scenario L which simulates increased growth due to climate change, projects the largest net forest carbon sink. In this scenario, the projected change in climate will improve the forest growth conditions on an aggregate EU level [20]. It follows that this scenario leads to the largest estimate of the net forest carbon sink. This finding is consistent with previous projections for individual EU Member States [21] and studies showing that environmental changes during that last decade have increased the net forest carbon sink [22]. However, it should be noted that our simulations do not consider potential changes in the occurrence or the severity of natural disturbances such as wildfire, windthrow and insect outbreaks.

It is important to note that the HWP carbon pool to a certain degree balances out the differences in the projected forest carbon sinks as seen between the different scenarios. This is particularly the case when comparing the differences between the scenarios A, B and L (see Fig. 2). While scenarios A and B project the smallest estimate of the forest land related carbon sink (i.e. above and below ground biomass, deadwood, litter, and soil), these scenarios at the same time have the highest estimates of the HWP carbon sink. This is to be expected as these scenarios have the highest forest harvest levels, which generally decreases the forest land related carbon sink and increases the HWP carbon pool (due to an increased inflow to this carbon pool). On the contrary, scenario L projects the highest estimate of the forest land related carbon sink, but a generally low estimate of the HWP carbon sink. This is then the opposite situation as to scenarios A and B, as in this case the scenario assumptions lead to a generally low future forest harvest level that result in a high estimate of the forest land related carbon sink and a generally low estimate of the HWP carbon pool.

### Regional impacts

**Fig. 3:**
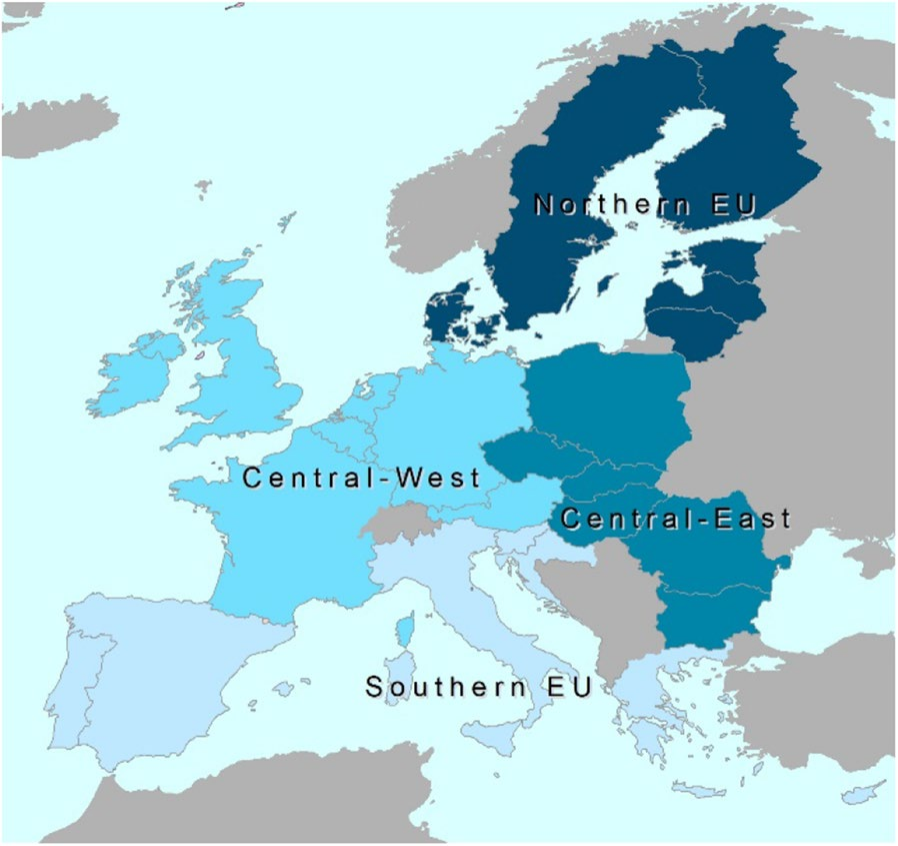
Regional division of the EU28 Member States as used for this assessment

**Fig. 4:**
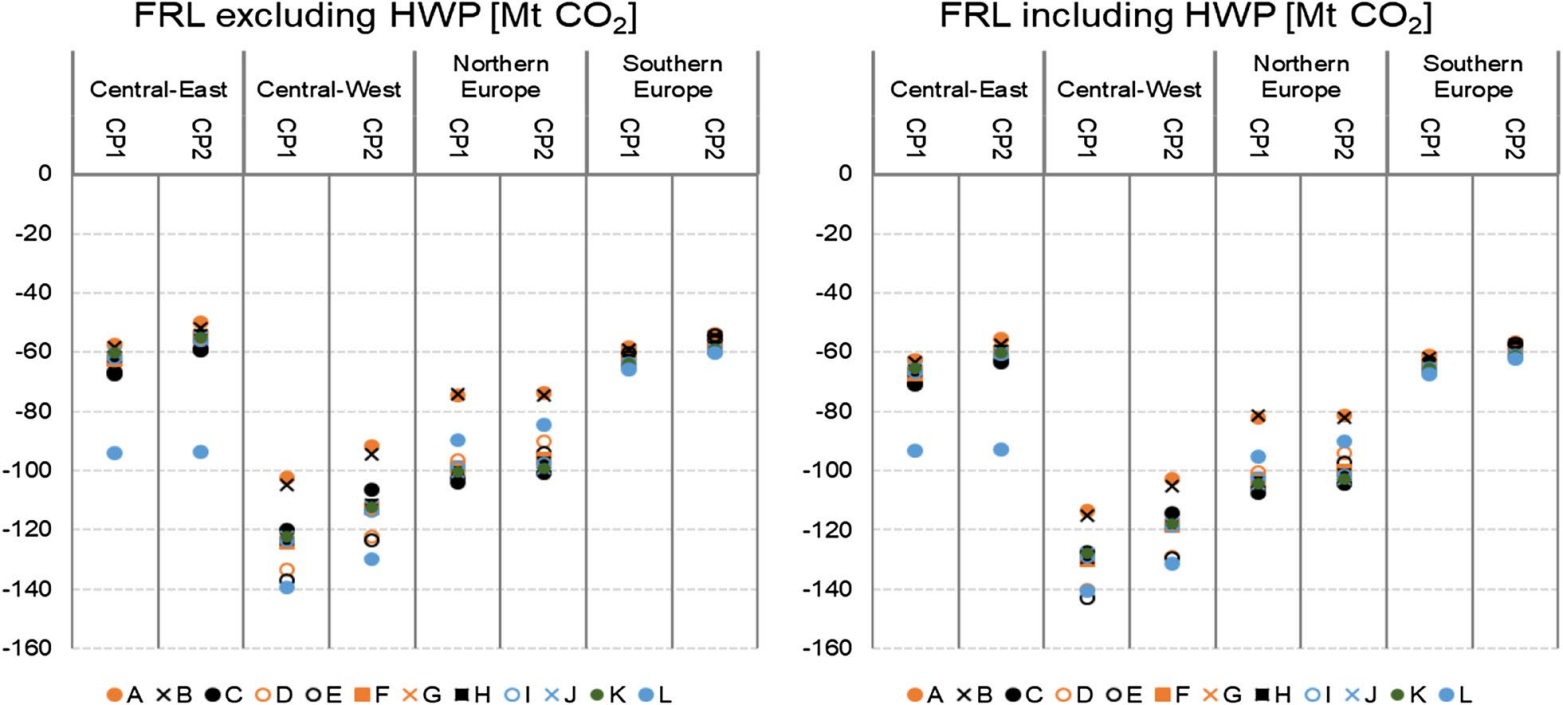
The aggregate FRL estimates for the four regions of Europe shown in terms of the FRL estimates for the first and second compliance period (CP1 and CP2, respectively), excluding (left) and including (right) the HWP sink

The projections of the 12 conceptual scenarios also show that there are notable differences in the regional implications of the different modelling assumptions. The regions assessed are shown in Fig. 3. Figure 4 shows the estimated FRLs for four regions of Europe, including and excluding HWP. Consistent with the aggregate EU28 results, the scenarios that project the smallest net forest carbon sink across the different regions are scenarios A and B. Furthermore, in three of the four regions (Central-East, Northern and Southern Europe) scenario L projects the largest net forest carbon sink. As for the EU28 aggregate results, the inclusion of the HWP carbon pool in the FRL estimate reduces the differences in the estimated FRL between the scenarios in all regions. Also, for each region the forest carbon sink is projected to be 5% to 10% smaller in the second compliance period than in the first compliance period, depending on the scenario. This result is consistent across the regions as well as with the aggregate EU28 results.

For Central-West and Northern Europe, the estimated forest carbon sink for the first and second compliance periods varies notably depending on the scenario assumptions taken. Depending on the scenario, the aggregated forest carbon sink (including HWP) for the first compliance period varies by 29 MtCO_2_ (−114 to −143 MtCO_2_) for Central-West Europe and by 26 MtCO_2_ (−81 to −107 MtCO_2_) for Northern Europe. However, for Southern and Central-East Europe (with the exception of scenario L) the forest carbon sink remains relatively stable between the different scenarios. Depending on the scenario, the aggregated forest carbon sink (including HWP) for the first compliance period only varies by 7 MtCO_2_ (−61 to −68 MtCO_2_) for Southern Europe, and by 8 MtCO_2_ (−63 to −71 MtCO_2_) for Central-East Europe (excluding scenario L). The large spread of the FRL estimates as noted for the regions of Central-West and Northern Europe is mainly related to the high temporal fluctuation of harvest rates during the reference period. In these countries, forests are used more prominently for industrial purposes, and the economic downturn of the end of the reference period affected the harvests more than in Central-East or Southern Europe. Especially, a large difference is noted between the harvest rate in 2009 and the average harvest rates for the reference period. The variability in the harvest rate during the reference period leads also to a relatively large range of estimates for the rotation time and area of land allocated between the different forest management practices. On the contrary, in Southern and Central-East Europe it can be noted that the historical harvest rates during the reference period was more stable, and thereby the different scenario assumptions have a smaller impact on the FRL estimates.

### Implications of different modelling assumptions for different Member States

To highlight the potential implications that different modelling assumptions may have on the projected national FRLs, we can assess the results of scenarios with regard to the differences in the underlying modelling assumptions. Table 3 shows how the 12 conceptual scenarios can be contrasted to each other to draw such lessons. Figure 4 shows the percentage change in the country-specific FRLs for the 28 EU Member States, depending on the type of assumption made. Comparing the outcome of the conceptual scenarios, two key insights can be drawn concerning the relevance and importance of different modelling assumptions for estimating the country-specific FRLs.

**Table 3:** Change in the FRL (including HWP) at the aggregate EU28 level measured as the difference in the estimated FRL between scenarios

	Alternatives (Alt) for the comparison	Scenarios used for comparison
Starting year of projections		
Starting projections in 2015 instead of 2010	Alt 1	A → B
	Alt 2	G → J
	Alt 3	H → K
Stratification of managed forest land		
No stratification according to tree species	Alt 1	F → D
No stratification according to MAI	Alt 1	F → C
No stratification according to tree species and MAI	Alt 1	F → E
Allocation of forest management practices		
Latest instead of average data sources used to allocate forest management practices	Alt 1	A → F
	Alt 2	B → I
Timing of management activities		
Latest instead of combined rotation time	Alt 1	H → G
	Alt 2	K → J
Average instead of combined rotation time	Alt 1	H → F
	Alt 2	K → I
Climate change		
Including consideration to climate change	Alt 1	I → L

Note that different ways that the scenarios can be contrasted to each other to draw lessons concerning a specific modelling assumption is here defined as an alternative (Alt)

**Fig. 5:**
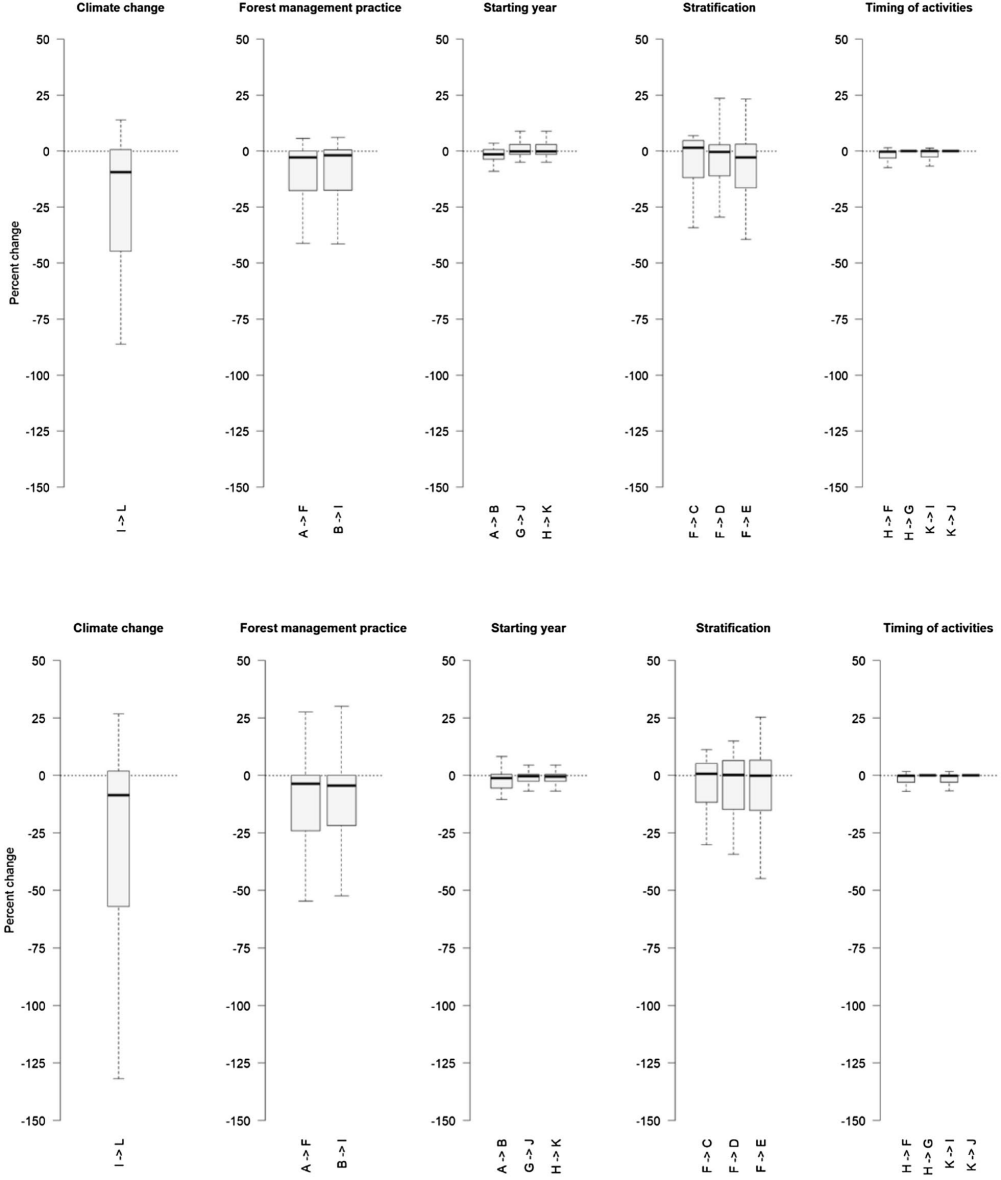
Percentage change of the country-specific FRL (including HWP) when comparing different scenarios. For each key assumption, percentage change is plotted for each of the 28 EU Member States. No weighting between Member States estimates is applied. Boxes represent the first to the third quartile range and the plain line indicates the median, dotted lines delineate the first and fourth quartile points up to 1.5 times the interquartile range of the box. The top figure shows the outcome for the first compliance period, and the bottom figure shows the outcome for the second compliance period. It should be noted that a negative percentage (“−”) here implies that the carbon sink in managed forest land is increasing, while a positive percentage (“+”) implies that the carbon sink is decreasing. For clarity, outliers are not represented in this figure

Firstly, assumptions related to the timing of management activities and the starting year of the projection have minor impacts on the estimation of the country-specific FRLs. This is the case as the dispersion around the median is small for the assumptions of timing of management activities and starting year of the projection (see Fig. 5). This result indicates that there are no large differences between the Member States related to the impact of these assumptions. This, as the timing of management activities only has a minor impact on the final harvest levels during the first and second compliance periods. The average harvest levels in the individual Member States fluctuate by less than 1% between the scenarios focusing on this specific assumption (i.e. scenario F, G, H, I, J and K). For the assumptions regarding the starting year, there is somewhat more variation between the Member States, but also this assumption does not in general have a large effect on the FRL estimate in the different countries. This is due to relatively small differences in the state of the forest (e.g. age structure) and forest management practices between 2010 and 2015, and also because most of the transition effect, caused by application of the reference period FMPs to the forest in the new state as of 2015, vanishes in first few years after starting the projection.

Secondly, assumptions related to climate change, allocation of forest management practices on the area of managed forest land, and the level of detail to which the area of managed forest land is stratified, have larger implications on the estimation of the FRLs and especially show a larger variation in the results across Member States (see Fig. 5). The large diversity in the impacts of changing assumptions related to climate change is not surprising given that, first of all, climate change patterns are different in different EU regions, and second, different tree species respond differently to the changing environment [20]. Moreover, the large diversity with regard to tree species, age structure and productivity of forests between the Member States, is also reflected in the rather large variation in the impacts of not stratifying the area of managed forest land according to tree species distribution and/or productivity classes. For the assumptions regarding the allocation of forest management practices, the considerable variation among the Member States reflects the variation of harvest levels during the reference period, which has a direct impact on the allocation of management with and without clearcutting in the G4M model. Higher harvest level is modelled through more clearcutting, while lower harvest level is modelled with relatively more forest management practices without clearcutting. As the EU Member States had different harvest patterns during the reference period, the impact of the assumption on how to allocate the forest management practices varies notably between the countries.

## Discussion

According to our estimates, the aggregate net carbon sink on managed forest land in the EU28 (including HWP) would be −319 to −397 MtCO_2_ during the period of 2021–2025, and −296 to −376 MtCO_2_ during 2026–2030, if the forest management practice of 2000–2009 was continued without changes. These results indicate a slight decrease of the EU forest sink in the future, which has been suggested also in other studies [7, 16, 18]. The variation between the estimates of this study reflects the range of impact due to different alternative assumptions that may be made in the estimation of forest reference levels in the EU under the new EU LULUCF Regulation [1].

In this study, we show that many of the studied assumptions, stemming from the details in the EU LULUCF Regulation and the related technical guidance document [8] allow for flexibility and do not have a strong impact on the forest reference level on the aggregate EU level. This is especially the case for assumptions such as the starting year of the projection for calculating the forest reference level, and the definition of the timing of forest management activities: our analysis shows a very small difference in the results between cases where the projection started in 2010 or 2015, and between cases where the timing of management operations (for example clearcut age) was determined based on the last year’s situation, or the average value during the reference period. On the other hand, there was more impact on the results of assumptions regarding the level of detail of the stratification of managed forest land, allocation of forest management practices, and especially in whether climate change impacts on forest growth were included in the projection or not.

The assumptions analysed in this paper are unavoidably model-specific for the G4M model, with the main impacts are rooted in silvicultural mechanics modelled by G4M. The noted strong impact of the assumptions related to forest management practices and the rather wide dispersion in the impact of these assumptions between different Member States is explained by the following. Forests with clearcutting are usually younger and contain less biomass than the forests without clearcutting. If the forest management practice is changed to one without clearcutting, the forest actively accumulates biomass (i.e. enhanced sink). Vice versa, if in a forest without clearcutting the forest management practice is changed to the one with clearcutting the forest loses biomass as it is intensively thinned, and a large share of older trees is removed according to the rotation period in the first decade (i.e. reduced sink). In countries with high annual variability of harvested wood during the reference period, the modelling assumptions regarding the area of forest used for wood production with clearcutting to be applied for the forest reference level projection may differ considerably (the case of the scenarios with “Average Allocation of FMPs” vs. the scenarios with “Last Allocation of FMPs”).

Furthermore, the starting year of the projection has little impact on the forest reference level because the duration of the transition process, caused by introducing the FMP parameters derived over the reference period into the model (dynamic system) in a new state, is short. Starting the FRL projection in 2015 causes a slightly greater distortion to the model than starting in 2010 as the FMP parameters determined in 2000–2009 are applied to the forest which has been developing longer, and therefore the distance in the parameter space between current parameters and the introduced parameters is larger. In general, the distortion is greater if the state of the forest and FMP’s (e.g. age structure, forest management practices with or without clearcutting, timing of forest management practices) in the projection starting year is further from the state of the forest and FMP’s in 2000–2009. The distortion causes a spike in forest management emission or sink and consequent damped oscillations. The closer the FRL projection start is to the compliance period the greater the impact of the initial spike and the consequent damped oscillations on the FRL is. Potentially, the FRL starting year could have a more noticeable impact on the FRL estimate if the FRL projection was started within 1–3 years before the commitment period.

The inclusion of climate change impacts on forest growth (scenario L) was found to have a clear impact on the results. It needs to be emphasized that the climate modelling results employed in this study are uncertain, and do not take into account for example the possible increase of natural disturbances that may be associated with climate change. Increased natural disturbances such as droughts, wildfires and insect outbreaks could counteract the possible positive development of the forest sink considerably, as suggested by e.g. Seidl et al. [23] and Hanewinkel et al. [24]. Therefore, our results should not be considered as an assessment of the possible carbon sink in the future under climate change, but rather as an example of a possible—and possibly notable—source of uncertainty in the FRL estimates. If the climate change impact is modelled in the FRL, and the realized impact during the CP will be according to this estimate, accounting against the FRL will cancel the impact of climate change, and reflect only changes in forest management practice. However, if there are climate change impacts assumed in the FRL that do not materialize under the CP, these assumptions could be falsely accounted for as the impacts of changed management between the RP and the CP. Therefore, if climate change assumptions are included in the FRL projection, the realized climate change impact should be compared with the projected ones before accounting for the emissions and removals against the FRL.

The results of this study also show clearly that the considerable diversity among the EU Member States makes the impacts of especially certain assumptions to vary between different countries. Given that the forest reference levels are estimated individually by each EU Member State, the results of this current study indicate only the possible uncertainty of the estimates on the aggregate level and cannot be used to estimate the uncertainty for any specific Member State. Moreover, the variety of forest models used within the EU also means that there is likely a plethora of different assumptions and interpretations of the Regulation being adopted by the Member States, and the full range of the aggregate forest reference level in the EU may in reality lie outside the estimates of this study. We note especially that care should be taken when comparing the estimates as provided in this study and with the final estimates as developed by the EU Member States and reported in their National Forestry Accounting Plan. As different models may be applied, methodological assumptions may differ and the input data sources as applied may not be the same. A large range of different models are now readily available and may be employed to project the development of forests [25]. How large differences in the estimation of the FRL will be due to the use of different models will of course vary depending of the inherent difference in the models themselves. However, the experience from the development of the earlier forest management reference levels has shown that for certain Member States, this difference may be minor [14].

Data sources describing the state of the forest at the beginning of the model run (e.g. total area of managed forest land, increment, biomass, and age-related information) may also very well differ between estimates and influence the estimated forest reference level. Most EU Member States have up-to-date information from national forest inventories that can be used to meticulously define the state of the forest and the description of the current forest management practices, thereby providing an accurate estimate of the forest reference level. Such information is crucial for these types of modelling assessment as it has been shown that underlying assumptions concerning the initial age class distribution, management activities, growing conditions and historical natural disturbances influence the projected amount of harvest considerably, and as a consequence the future emissions and removals from managed forest land [21, 26]. Further work on this subject would be useful not only to compare the outcome between models that are inherently different (i.e. stand level models vs. regional models), but also dependencies on the actual data sources being applied for developing the projections.

## Conclusions

This study provides a first assessment of how different modeling assumptions arising from the flexibility in the LULUCF regulation and the technical guidance document may influence the country-specific forest reference levels, and what the effect of this impact is on an aggregate EU28 level. Applying the interlinked G4M and WoodCarbonMonitor modelling frameworks, we estimate country-specific forest reference levels covering all carbon pools covered by the EU LULUCF regulation (i.e. above-ground biomass, below-ground biomass, litter, dead wood, soil organic carbon and HWP) for a set of conceptual scenarios, each scenario have been developed in accordance with the guidance as provided in the LULUCF regulation and the technical guidance document.

As different underlying assumptions may lead to differences in the estimated forest reference level, these results highlight the importance of transparent documentation by the EU Member States on how their forest reference level has been calculated, and what the underlying assumptions are. As one of the key aims of accounting rules defined in the LULUCF regulation is to provide a solid framework for comparable standards, it is vital to know how each Member State has performed their calculation and what assumptions they have applied. Without transparent documentation, there is a risk that the efforts of the Member States in maintaining and enhancing their LULUCF sinks are not accounted for properly. After all, the forest reference level provides only a counterfactual value for the accounting of emissions under the compliance period. The real challenge—and opportunity—will be to enhance the forest sector’s role in contributing to climate change mitigation. This requires a careful consideration of the trade-offs associated with the different possibilities of forest management to affect carbon sequestration. A combination of preserving forest carbon stocks, enhancing forest growth through management, substitution of fossil feedstocks with renewable materials, and prevention of natural disturbances is needed. To have a real impact on climate change and a credible role in decision making, it is essential to account for all the efforts in a reliably and exhaustively.

Based the LULUCF regulation that has been agreed upon, it is now the responsibility of each individual Member States to contribute to the integrity of the accounting system and ensure development of a forest reference level that considers national circumstances and that is consistent with the regulation. As forest management may play a key role in meeting climate targets of Nationally Determined Contributions under the Paris Agreement, it is vital that the EU and its Member States carry the responsibility to properly account for emissions and removals and to ensure the integrity of the jointly agreed upon accounting system.

## Methods

### The G4M model

The Global Forest Model (G4M) is applied and developed by IIASA [9, 27–30]. The model estimates the impact of forestry and land use change activities (forest management, afforestation and deforestation) on biomass and carbon stocks. The model is geographically explicit and can be applied to estimate the carbon impact based on external information (projections of wood demand, wood price and carbon price), different management activities (e.g. rotation period, thinning intensity, tree species), and differences in income from alternative land use on the same place. Decisions concerning forestry and land use change activities are calculated for 0.5 × 0.5° grid cells, which approximately corresponds to a 50 × 50 km grid taking sub-grid information into account as described by Gusti and Kindermann [9] and Gusti et al. [28]. G4M produces estimates of forest area change, carbon sequestration and emissions in forests, impacts of carbon incentives (e.g. avoided deforestation, afforestation, improved forest management) and supply of biomass for bio-energy and timber.

The main forest management options considered by G4M are adjustment of rotation time and variation of forest area from which wood is harvested in a sustainable manner [27].^3^ Management options can be individually selected by the model and can estimate optimal rotation lengths to maximize increment, stocking biomass or harvestable biomass. Increment is determined by a potential Net Primary Production (NPP) map [31] and translated into Mean Annual Increment (MAI). During a simulation, increment observed in a particular year is adjusted over time based on changes in the age structure, stocking degree, and environmental information (i.e. temperature, precipitation and CO_2_ concentration).

For this study, the model is first run to simulate historical wood production in the EU countries in 2000–2009 or 2000–2014 (depending on the assumed starting year of the FRL projection), while the rotation time and allocation of forest management practices are recorded for each year of the reference period 2000–2009. The state of the forest (age structure, age related biomass, diameter and height) is recorded at the end of the “historical” period (i.e. in 2009 or 2014) in each grid cell. The recorded rotation time and the forest management practice are then used for determining the management options to be applied for the FRL projections according to the considered scenarios (see Table 1). The recorded state of the forest is used for initialising the model for the FRL projections.

For each prospective scenario, two key pieces of information are passed on from G4M to the WoodCarbonMonitor model: (i) the amount of felled or otherwise harvested and removed roundwood from managed forest land for the period 2000–2030 as estimated by G4M, (ii) the amount of felled roundwood from managed forest land converted to other land uses for the period 2000–2009 as estimated by G4M.

### The WoodCarbonMonitor model

The WoodCarbonMonitor, developed by Rüter [12], is a model that estimates GHG effects from HWP based on the annual carbon inflow to the carbon pool in HWP. It constitutes a development of the IPCC HWP model and is integral part of the German national GHG reporting framework for the land use sector. It has already been applied for estimating the HWP contribution to FMRL of several European countries during the second commitment period of the Kyoto Protocol [15] and enables estimating historical as well as projected GHG emissions and removals associated with harvested wood.

The model implements different reporting approaches presented by the IPCC (i.e. stock-change, production and atmospheric flow approach) and further methodological elements provided in the latest IPCC guidance [32]. This also permits tracking and differentiating between the carbon impacts in relation to the origin of the wood harvest, i.e. the products’ associated land-use categories or forest related activities as well as the woody feedstock contained in the relevant HWP commodities (e.g. industrial roundwood, pulp and recovered paper). The model furthermore includes detailed and representative information from life cycle assessment (LCA) information for all relevant HWP commodities [33, 34]. Besides its integrated interface to the G4M model, the WoodCarbonMonitor may also apply projected harvest and traded HWP commodities estimated by the GLOBIOM model [35].

The WoodCarbonMonitor covers all relevant HWP commodity data of all 28 EU member states and major HWP producing countries as included in FAOSTAT [19]. Further country-specific information e.g. on further processing of semi-finished wood products to finished products and/or their use in different market sectors (e.g. for the building sector) allows for estimating country- and case specific decay patterns and GHG implications associated with the use of harvested wood [36].

### Parameterization of models to national specific circumstances

For this assessment, the forest area in G4M was set to match the reported forest area in 2000 according to Forest Europe [37]. The initial forest growing stock (aboveground biomass) per grid cell is taken from the European forest biomass map by Gallaun et al. [38]. A total of eight prevailing tree species groups are considered in the G4M model, namely: fir, spruce, pine, aleppo pine, birch, beech, oak and larch. Forest growth function for each of these major tree species groups are defined in G4M according to forest growth functions developed by Kindermann et al. [20]. Tree species distribution in each grid cell is distinguished using a species map by Brus et al. [39] by aggregating the original raster 1 × 1 km into the G4M 0.5 × 0.5° grid cells using the “majority” option. Therefore, the one species occupying the largest share of the 0.5 × 0.5° grid cell is assigned to represent the specific cell. The growing stock obtained from the Gallaun et al. [38] map is scaled to data reported to Forest Europe [37]. For further initialisation of the forest state the model uses forest age class structures for countries as in the study by Böttcher et al. [14]. Historical wood harvest data for the period of 2000 to 2009 or 2014 (depending on the assumed starting year of the FRL projection) are taken from FAOSTAT [19]. Spatial allocation of harvest within each Member State is initialized using a map of 2000–2010 average harvest by Verkerk et al. [40]. Area of managed forest land is assumed to remain constant from the starting period of the starting year of the FRL calculation, in-line with the alternatives specified in the technical guidance document.

The G4M model may also apply projections of wood demand for Member States (as for example estimated by the GLOBIOM model [41]) to endogenousely estimate forest management decisions and wood production activities. In such a case, G4M simulates forest management decisions for every forested grid cell within a Member State to match the exogenous wood demand provided at the national level. The initial forest management practices for each grid cell are estimated based on the wood production map and the wood demand on the national scale and the forest characteristics in the grid cells, while the changes of the forest management practices in the consequent years are driven by the dynamics of the wood demand and comparison of the net present values of forestry for the current and new forest management practices. The new forest management practice for the forest in the grid cell is accepted if it decreases the gap between the wood production and the demand [9, 27], and it does not decrease the net present value of forestry comparing to the current one.

G4M distinguishes three general categories of forest management practices: (i) timber production forest with clearcutting (thinning and clearcutting are being applied), (ii) timber production forest without clearcutting (continuous forest cover where only thinning and selective logging can be applied), and (iii) protected forest with no harvesting (e.g., nature reserve, wilderness area, national park etc.). We assume that the area of timber production forest with and without clearcuttings can change over time depending on wood demand [27], while the area of the protected forest is initialized following the World database on Protected Areas [42] and remains fixed over time. For the FRL simulation the area of production with and without clearcuttings changes only during the historical period (until the beginning of the FRL projection) and is fixed throughout the two compliance periods; i.e. no assumptions on the development of future wood demand were taken. The allocation of the two forest management practices for the FRL is estimated based on the reference period, depending on scenario-specific modelling assumptions (for details see “Allocation of forest management practices to the area of managed forest land” section).

For estimating the HWP implications of EU in the FRL, the production approach as described by IPCC [32] on the basis of latest FAO data is implemented. As described in Forsell et al. [8], only timber production associated with the land use category Forest Land remaining Forest Land as calculated by G4M has been considered. As a consequence, HWP originating from managed forest land converted to other land uses (deforestation) is not included in the FRL.

### Scenarios for projecting the forest reference levels

For this assessment, a total of 12 conceptual scenarios were developed to assess the potential impact of different assumptions on the projection of the country-specific FRLs.

#### Starting year for the projection of the FRL

The LULUCF Regulation does not explicitly state from which year the projection of the FRL should be started: the modelling could therefore start right after the reference period, or for example from the year when the FRL is estimated (i.e. 2018). The technical guidance document states that as a default, it is good practice that Member States start the projection of the FRL as of 2010 or earlier. Consequently, 2010 would be the first year that the modelling framework projects the development of forests on managed forest land. However, a Member State may select a different starting year for the projection of the FRL if justified.

Two potential starting years for the FRL projection are considered for this assessment, 2010 and 2015. In both cases, the G4M model endogenously allocates the forest management practices and the timing of management activities for each grid cell until the year preceding the selected starting year of the FRL. From the starting year onward, the allocation of forest management practices, and timing of when the individual management activities are to be carried out, are set for each grid cell according to the scenario-specific assumptions and kept constant over time, as in the FRL these are not driven by an exogenously defined wood demand. Area of managed forest land is assumed to remain constant from the starting year of the FRL projection, in-line with the alternatives specified in the technical guidance document.

#### Stratification of managed forest land

Stratification of the managed forest land is a concept that is being used in the technical guidance document [8], but it is not explicitly mentioned in the LULUCF regulation. In the technical guidance document, stratification is used to divide the managed forest land into classes that can be connected to certain types of forest management practices. This stratification can be used as a basis for the allocation of the forest management practices. To comply with the requirement of continuation of historical forest management practices, the criteria used for stratification are to remain the same in the modelling of historical and projected emissions and removals. Numerous criteria for stratifying the MFL are described in the technical guidance document [8, Section 2.1]. Here, we assess the application of four different criteria for the stratification.

In the case of the Full assumption, each EU Member State is stratified to as detailed extent as feasible utilizing the G4M model, mimicking the case where the Member States apply detailed stratification. In this approach, the managed forest land is stratified in G4M according to two main criteria: (i) the eight main tree species groups covered by G4M, and (ii) site productivity classes (as determined by bio-geographical site conditions).

With 1Species assumption, the stratification mimics a case when a Member State selects not to perform the stratification according to different tree species groups. This represents a case where, for example, the Member State does not have sufficient information to differentiate management practices between different tree species or geographic information about the location of the different tree species. In this approach, stratification is still performed according to site productivity. However, for each Member State, a single prevailing tree species group is selected for the Member State (based on the most abundant tree species in the Member State) and this species group is assumed to be prevailing in all grid cells associated with the Member State.

In the case of the 1MAI assumption, the stratification mimics a case where a Member State selects not to perform the stratification according to site productivity classes. In this approach, stratification is still performed according to tree species. However, no stratification is done according to site productivity that is represented by MAI in the model, therefore it assumes that one average MAI is implemented in the assessment of the FRL for the whole Member State.

In the case of the 1MAI and 1Species assumption, the stratification mimics a case where a Member State selects not to perform the stratification according to productivity classes nor according to tree species groups. In other words, this forms the case where the Member State does not have sufficient information to define differences in management practices neither between tree species nor site productivity classes. In this approach, all the forests within the Member State are treated homogenously based on a single uniform descriptive site productivity and tree species (i.e. a combination of the 1Species and 1MAI assumptions as detailed above).

#### Allocation of forest management practices to the area of managed forest land

In the same way that the criteria used for stratifying the area of managed forest land are advised in the technical guidance document to be kept constant throughout the calculation of the FRL, the forest management practices assigned to the forest are also advised to be kept constant throughout the compliance period. In G4M, the FRL is calculated based on the delineation of the area of managed forest land according to three main categories of forest management practices: (i) timber production forest with clearcutting, (ii) timber production forest without clearcutting, and (iii) protected forest without harvests (e.g., nature reserve, wilderness area, national park). Two different assumptions are here considered regarding data used for allocating the area of forests used for timber production with and without clearcuttings.

In the case of the Average assumption, the forest management practice in each grid cell of the G4M model is determined based on the majority of years, during which the practice was applied within the historical period 2000 to 2009. As an example, if in 6 out of 10 years in 2000–2009 the forest in the cell was managed with the management practice including clearcutting, this practice is consistently applied on that area throughout the compliance period. In case of equal number of years when a particular practice was applied, the forest management practice being applied in 2009 determines the decision.

In the case of the Last assumption, the forest management practice applied in 2009 is assumed to be continued to be implemented throughout the compliance period. In other words, the forest management practice applied as of 2009 in each cell in G4M is consistently applied throughout the first and second compliance periods.

In both cases, the area of land allocated to each forest management practice remains constant from the starting year of the projection. Furthermore, the area of protected forest is independent of these scenario-specific assumptions as it is always initialized following the WDPA [42] dataset and remains and the same for the Average and Last scenario.

#### Timing of individual management activities

A key criterium for the FRL is that it “*shall be based on the continuation of sustainable forest management practice, as documented in the period from 2000 to 2009*” [1, Art 8(5)]. However, the timing of when the individual management activities are to be implemented need to be defined in such a manner that a modelling framework can apply these activities for the FRL estimations. To address this question, three different assumptions are considered, all directly related to the rotation period applied in the grid cells in G4M throughout the first and second compliance periods. For grid cells without clearcutting, rotation period is not applicable and therefore they are modelled similarly under each of the three assumptions.

In the case of Average assumption, the rotation period to be applied for each grid cell is calculated as the average for the same grid cell during the historical period 2000 to 2009 (if the forest management practice allocated to the grid cell was timber production with clearcutting). In other words, the rotation period for each grid cell in G4M is calculated as the average for the period of 2000 to 2009 (i.e. the reference period), and consistently applied throughout the first and second compliance periods.

In the case of Latest assumption, the latest documented rotation period (in case of clearcutting) as of 2009 is assumed to be continued throughout the first and second compliance periods. In other words, the rotation period as of 2009 is continued for each cell in G4M and consistently applied throughout the first and second compliance periods.

In the case of Combined assumption, a combination of Average and Latest is applied across the Member State. For each individual grid cell, we check if there is a trend in the length of the rotation period during the reference period (e.g., a longer or shorter rotation period applied in 2009 as compared to 2000). This is done through by first calculating a least squares line for the rotation periods as implemented during the period 2000 to 2009. If the beginning and end of the trend line differ by more than 5%, it is assumed that the rotation time as of the year 2009 will continue to be applied throughout the first and second compliance periods. In other words, if a trend is observed for the rotation time, the same assumption as for Latest is assumed for the grid cell. If no such trend is observed for the grid cell, then the average rotation time during the historical period 2000 to 2009 is applied for the cell (i.e. same as for Average).

#### Assumptions concerning climate change

The estimation of the FRL may be impacted by changes in climatic conditions such as changes in precipitation, temperature, and CO_2_ and nitrogen deposition feedbacks. Depending on the type of modelling framework applied to estimate the FRL, different assumptions concerning future climatic conditions and the related carbon impacts for the projection of the FRL can be taken. The technical guidance document advises to clearly document whether or not the FRL includes modelling of climate change-related impacts on forests, and what the related assumptions are. In this paper, we estimated the FRL without and with assumptions on climate change impacts on forests.

In the case where no considerations to future climate effects are considered (i.e. No assumption), it is assumed that the climatic conditions will not change during the first and second compliance periods (i.e. the impact of climate on the forests is assumed to stay constant over time). In this case, the same climatic conditions as for a historical time period are being used for the compliance periods. This assumption depicts a case where the modelling framework applied to project the FRL is not able to consider changes in climatic conditions, or where the Member State deems that the uncertainty associated with climate change modelling is too high to produce reliable estimates.

In the case where changes in climatic conditions are considered (i.e. Yes assumption), it is assumed that the future climatic conditions are known, and the impacts of the related changes are accounted for in the projection of the FRL. For this analysis, the response of tree growth for the eight prevailing tree species groups as considered in the G4M are set according to climate projections for the SRES A1b emissions scenario from Kindermann et al. [20], with spatially explicit growth response for the different tree species across Europe. According to this climate scenario, the radiative forcing is close to RCP8.5 by 2050 but then it declines and is between the RCP6.0 and RCP8.5 by 2100 [43]. The model assumes no effect of CO_2_ or nitrogen deposition feedbacks. In this paper, we apply the 2021–2030 average change in MAI relative to the 2001–2020 average, as determined in Kindermann et al. [20], to inform G4M of the MAI variation due to the climate. The relative change of MAI is derived from NPP averaged over the results of three models (Picus, Prelued and Gotilwa+) as presented in Kindermann et al. [20]. It should be noted that in this case, only changes in growing conditions are considered and that no adaptation of tree species to changes in climate conditions are considered nor accounted for.

## Data Availability

The data supporting our conclusions are either in the paper itself or in the links listed in the references. Additional data may be requested from the corresponding author.

## Abbreviations

CP: compliance period
ETS: emissions trading system
ESR: effort sharing regulation
EU: European Union
FMRL: forest management reference level
FRL: forest reference level
G4M: the global forest model
GHG: greenhouse gas
HWP: harvested wood products
LULUCF: land use, land use change and forestry
MAI: mean annual increment
MFL: managed forest land
RP: reporting period
UNFCCC: United Nations Framework Convention on Climate Change

## Footnotes

1 It should be noted that the forest sink is here represented with a “−” sign to clarify that the removals are larger than the emissions. Also, the more negative the number is, the greater the sink is.

2 In Nabuurs et al. [18], stem volume increment is defined as the average annual volume of gross increment minus average annual volume of natural mortality of tree, but including all stem volume harvested or affected by natural disturbances using the minimum diameters defined for growing stock.

3 Sustainable harvest is here interpreted that the harvest is not exceeding the increment.
